# A Highly-Ordered 3D Covalent Fullerene Framework**

**DOI:** 10.1002/anie.201411344

**Published:** 2015-05-08

**Authors:** Norma K Minar, Kun Hou, Christian Westermeier, Markus Döblinger, Jörg Schuster, Fabian C Hanusch, Bert Nickel, Geoffrey A Ozin, Thomas Bein

**Affiliations:** Department of Chemistry and Center for NanoScience (CeNS)University of Munich (LMU), Butenandtstrasse 5–13, 81377 Munich (Germany); Department of Physics and Center for NanoScience (CeNS)University of Munich (LMU), Geschwister-Scholl-Platz 1, 80539 Munich (Germany); Department of Chemistry, University of Toronto80 St. George Street, Toronto, ON, M5S 3H6 (Canada)

**Keywords:** covalent frameworks, electron mobility, fullerenes, mesoporous materials, self-assembly

## Abstract

A highly-ordered 3D covalent fullerene framework is presented with a structure based on octahedrally functionalized fullerene building blocks in which every fullerene is separated from the next by six functional groups and whose mesoporosity is controlled by cooperative self-assembly with a liquid-crystalline block copolymer. The new fullerene-framework material was obtained in the form of supported films by spin coating the synthesis solution directly on glass or silicon substrates, followed by a heat treatment. The fullerene building blocks coassemble with a liquid-crystalline block copolymer to produce a highly ordered covalent fullerene framework with orthorhombic *Fmmm* symmetry, accessible 7.5 nm pores, and high surface area, as revealed by gas adsorption, NMR spectroscopy, small-angle X-ray scattering (SAXS), and TEM. We also note that the 3D covalent fullerene framework exhibits a dielectric constant significantly lower than that of the nonporous precursor material.

An entirely new branch of chemistry was developed after the discovery of fullerenes in 1985 and after access to C_60_ on a preparative scale was gained in 1990.[1] The structural uniqueness of the C_60_ molecule sparked the interest of materials scientists to use it as a building block for novel materials with intriguing properties.[2] Versatile two-dimensional and three-dimensional exohedral modification options (i.e. a modification outside the spherical molecule) stem from the multifunctionality of the fullerenes, thus making them attractive precursors for macromolecular and supramolecular chemistry.[2]–[3] Fullerene polymers are being developed to integrate the intriguing properties of C_60_ molecules with the good processability and excellent mechanical stability of polymers.[4]

The most straight-forward approach to obtain a polymer using solely C_60_ molecules is photopolymerization. The resulting fullerene polymer is nonsoluble, stable, and highly crosslinked, but it is disordered and no control over the resulting structure is possible.[5] More sophisticated strategies polymerize the C_60_ or functionalized fullerene derivatives with the addition of auxiliary monomers to incorporate the fullerene core into a polymer chain.[6] By using this method, however, only a few weight percent of fullerene functionalities can be introduced into the polymer chain.[7] In a different approach, a star block polymer with a C_60_ core was designed to self-assemble into different thin-film structures ranging from lamellar to gyroidal, but without any intermolecular connection.[8] Other researchers have crosslinked a surfactant-like fullerene derivative that resulted in a 2D structure.[9] An alternative approach with metal-coordinated connections in between the fullerene derivatives produced 2D-layered structures with very small pores.[10]

Despite the synthetic efforts in the field of fullerene polymers and other fullerene-based materials, to our knowledge no examples exist of covalently crosslinked fullerene materials with three-dimensional order and stable high porosity. The introduction of ordered porosity is expected to create additional desirable features, such as high surface area and molecular discrimination, that would be beneficial for catalytic applications or electronic interactions of the fullerene pore-wall material with molecular guests.[11]

Herein we demonstrate the first example of a stable covalent fullerene framework exhibiting a highly-periodic 3D pore system with around 7.5 nm pore diameter. This high porosity was achieved by developing an evaporation-induced self-assembly (EISA) strategy of a fullerene precursor templated by a liquid-crystalline block copolymer inducing high periodicity and porosity. In this context, fullerene molecules can be modified at many points on their surface, potentially resulting in a multitude of adducts with different symmetries and a varying number of functionalities. This would lead to a complicated coassembly behavior between the precursor and template, and likely produce limited order in the final product.

We surmised that a hexafunctionalized C_60_ derivative with *T_h_* symmetry would be beneficial for the construction of a well-defined highly-ordered three-dimensional porous framework. The resulting covalent fullerene framework could show extraordinary thermal stability and interesting electrical properties.

The building block for the 3D covalent fullerene framework—a molecular hexafunctionalized fullerene—was synthesized by applying the template-directed activation method developed by Hirsch et al. (Scheme 1).[12] We successfully synthesized and purified the resulting hexa-adduct with a *T_h_*-symmetric octahedral addition pattern. The ^13^C NMR spectrum of the product demonstrates this high symmetry and the purity of the precursor (see the Supporting Information, Figure S1).

**Scheme 1:**
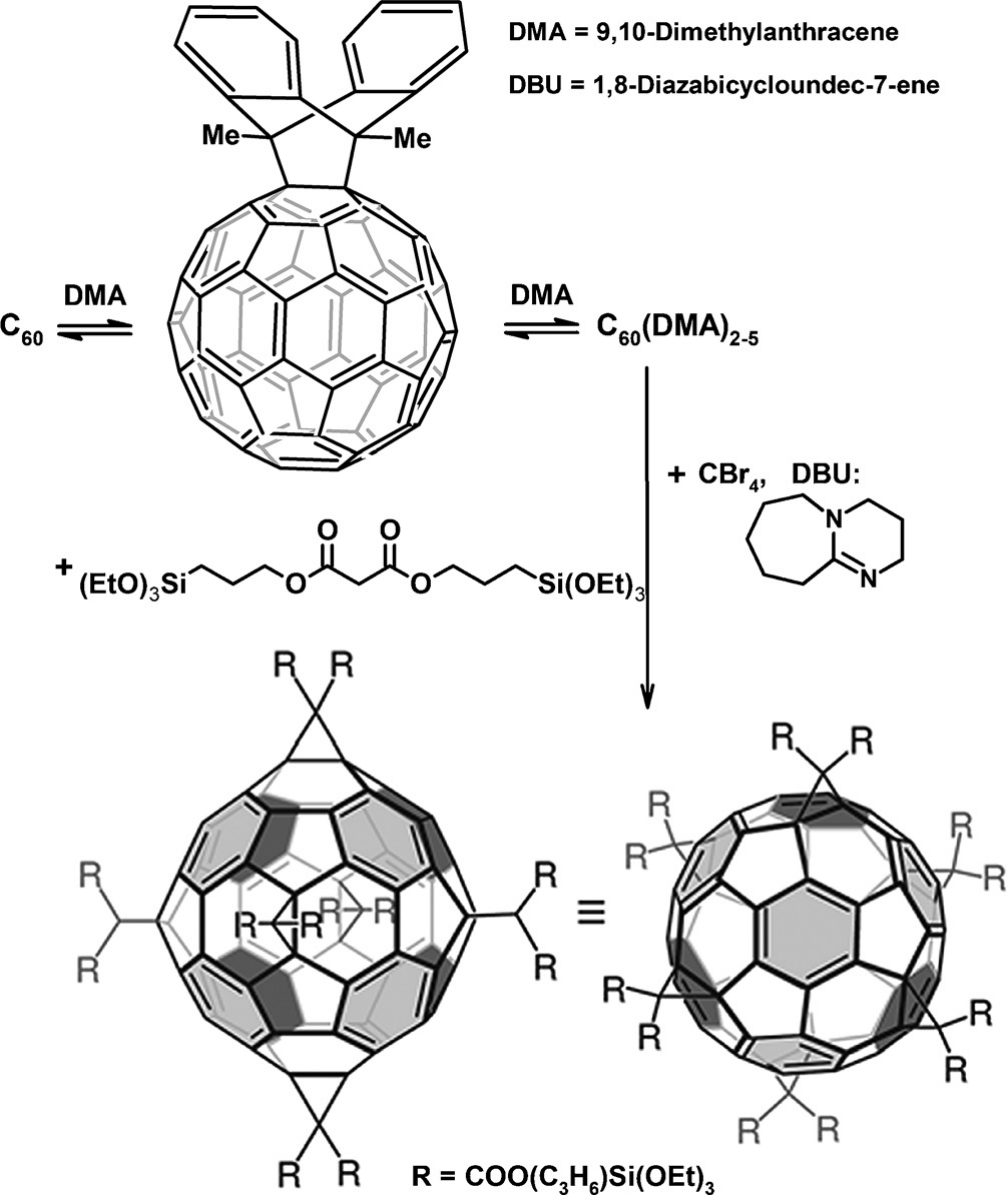
Synthesis of the hexafunctionalized fullerene by DMA templating and further cyclopropanation with silane malonates.

Thin films of the fullerene framework were produced in an EISA process by spin coating an ethanol solution of the hexafunctionalized fullerene and a block copolymer as template onto different substrates, such as glass or transparent conductive oxides. The resulting films proved to have a highly ordered mesostructure as demonstrated by means of small-angle X-ray scattering (SAXS), as shown in Figure 1.

**Figure 1:**
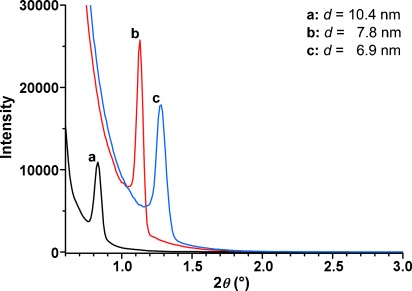
SAXS patterns of thin films of a highly ordered fullerene framework showing patterns for a) the as-synthesized film, b) the film after 18 h at 100 °C and solvent extraction, and c) the film after 18 h at 100 °C, solvent extraction, and 1 h under a nitrogen atmosphere at 300 °C.

For the as-synthesized film, a narrow reflection with a full width at half maximum (FWHM) value of 0.05° was detected at 0.85°, indicating a highly-ordered structure with a *d* spacing of 10.4 nm along the film normal. After a heat treatment at 100 °C and template extraction with ethanol, the *d* spacing decreased to 7.8 nm. Likewise, after a second thermal treatment at 300 °C for 1 h under a nitrogen atmosphere, the *d* value decreased further to 6.9 nm. This is in good agreement with the typically reported uniaxial shrinkage of mesoporous thin films along the substrate normal.[13]

Figure 2 shows TEM micrographs of fullerene-framework films after thermal treatment at 300 °C recorded in cross section and plan view, that is, perpendicular to and along the substrate normal, respectively. The cross section (Figure 2 a–c) shows *d* values of 11.0 nm parallel to the film and 6.8 nm along the film normal, which is in good agreement with the values determined by SAXS. Plan-view images (Figure 2 d–f) show mutually rotated periodic domains extending over large areas in the μm range. The domains show a rectangular lattice with *d* values of 11.0 nm and 7.9 nm. The ratio of these values (1.39) and the *d* value along the film normal of 10.4 nm before further treatments indicate an initially cubic structure with space group *Im*$\bar 3$
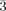
*m* and [011] orientation along the substrate normal. Assuming a lattice constant of 15.7 nm, the observed *d* values viewed along the [011] direction of such a structure are *d*_(0-11)_=11.1 nm and *d*_(200)_=7.9 nm, which fits well with our TEM observations. Along the film normal, the slight deviation of the value expected for *d*_(011)_ (10.4 nm instead of 11.1 nm) observed after film synthesis can be explained by a slight uniaxial shrinkage during drying. As a result of the shrinkage after heat treatment, the structure becomes orthorhombic, with space group *Fmmm* and lattice basis vectors **a**_orh_=**a**_cub_, **b**_orh_=(**b**_cub_+**c**_cub_)×*S*, and **c**_orh_=**c**_cub_-**b**_cub_, where *S* is the shrinkage factor. In the orthorhombic setting, the indexing of the observed lattice planes changes as follows: (0$\bar 1$
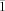
1)_cub_ becomes (002)_orh_ and (011)_cub_ becomes (020)_orh_. The relationship of the initial cubic structure and the orthorhombic structure is depicted in Figure S4. Films with the same space groups and orientations with respect to the substrate exist for carbon and metal oxides.[14] The symmetry change of cubic films with *Im*$\bar 3$
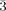
*m* symmetry in [011] orientation along the film normal to orthorhombic *Fmmm* in [010] orientation is discussed in detail by Falcaro et al.[13a]

**Figure 2:**
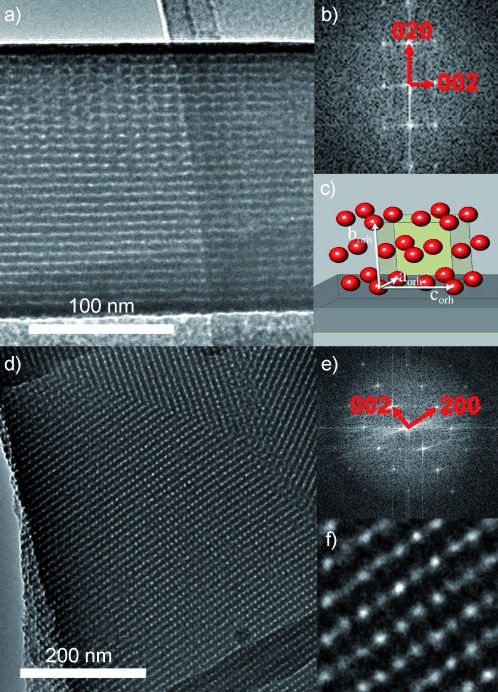
TEM images of the fullerene-framework film (190 nm thick) thermally treated at 300 °C under N_2_ for 1 h. a) Cross-section image, viewed along the [100] direction, of the orthorhombic structure with *Fmmm* symmetry. The lattice planes perpendicular and parallel to the substrate with measured distances of 6.9 and 11 nm can be indexed as (020) and (002), respectively. b) 2D Fourier transform of the TEM image in (a). c) Representation of the orientational relationship of the orthorhombic structure and the substrate viewed along [100]. d) Plan-view image in [010] orientation, showing large, highly ordered domains. The *d* values of 11 nm and 7.9 nm are in good agreement with the lattice plane distances of (002) and (200), respectively. e) 2D Fourier transform of the largest domain of (d). f) Expanded version (5×) of the TEM image in (d) on the left side.

The local chemical structure of the fullerene framework was examined by ^13^C and ^29^Si solid-state NMR spectroscopy (Figure S3 A). The ^13^C cross polarization magic-angle spinning (CP-MAS) NMR spectrum of the film removed from the substrate corresponds very well with that of the fullerene precursor, which confirms the integrity of the molecular structure of the precursor in the fullerene framework (see the Supporting Information, Section 4). The clear absence of *Q* units [*Q*^*n*^=Si(OSi)_*n*_(OH)_4-*n*_] at around *δ*=−100 ppm in the solid-state ^29^Si MAS NMR spectra (Figures S3 B, C) shows that there is negligible hydrolytic Si=C bond cleavage and that the siloxane-bridged organic linkers are retained intact in the fullerene framework under the synthetic conditions employed.

Thermogravimetric measurements give additional evidence that the fullerene framework, and especially the molecular structure, are stable in nitrogen up to 300 °C (Figure S5).

The accessible porous structure of the fullerene framework after template removal by solvent extraction was examined by nitrogen sorption of the film material. As shown in Figure 3, the isotherm shows a typical type-IV shape, commonly found with mesoporous materials. The hysteresis indicates a structure with large, cage-like pores that is typical for cubic mesostructures.[14b]

**Figure 3:**
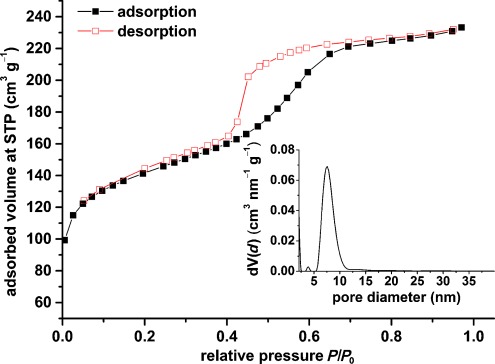
Nitrogen physisorption isotherms of the template extracted fullerene framework. Inset: the fitted pore-size distribution. STP= standard temperature and pressure.

The estimated pore-size distribution shows a sharp maximum at 7.5 nm (Figure 3, inset). The material shows a BET (Brunauer–Emmett–Teller) specific surface area of 494 m^2^ g^−1^ and a total pore volume of 0.34 cm^3^ g^−1^. We note that the internal voids of the fullerene moieties are not detected by the nitrogen adsorption measurements. Moreover, at the high-pressure end of the isotherm we observe a complete lack of textural porosity, which confirms the high crystallinity of this material. Additionally, at low pressure no discontinuity between adsorption and desorption branches is visible, implying quite a rigid open framework with essentially no swelling and shrinking.

The electronic properties of the fullerene-framework film were subsequently investigated. In contrast to the unfunctionalized fullerene, the hexafunctionalized fullerene shows nearly no light absorption above *λ*=300 nm (Figure S6).[15] This can be explained by the attenuation of the conjugated fullerene π-electron chromophore by virtue of transforming six double bonds into cyclopropane moieties.[16] The UV/Vis absorption spectrum of the fullerene-framework film resembles that of the precursor solution, with maxima at *λ*=238 nm and 275 nm (Figure S6). The absence of any shift for the porous structure indicates that the single fullerene precursor molecules are very well dispersed in the framework without electronic coupling and aggregation, similar to the situation in solution.[2] The absence of aggregation can be explained by the molecular structure of the precursor with *T_h_* symmetry, where the six malonate molecules are added to the fullerene cage in an octahedral pattern. This allows the fullerene molecules in the framework to be fully and omnidirectionally separated and thus electronic coupling can be avoided.

To probe the influence of the C_60_ side groups on electronic mobility, field-effect mobility measurements with bottom-gate and top-contact device configurations were performed. The transconductance characteristics of the monoadduct, the hexa-adduct without silane groups, and the final fullerene framework after template extraction are shown in Figure 4 a. During sweeps of the gate voltage at a constant source–drain voltage (*V*_SD_=20 V), the two hexa-adduct films do not show any significant current, whereas the monoadduct device shows the characteristic signature of an n-type semiconductor. An electron mobility of the order of 10^−4^ cm^2^ V^−1^ s^−1^ was measured for the monoadduct. The conductance curves (Figure 4 a, inset) for two constant values of the gate voltage (*V*_G_), one close to the threshold voltage (*V*_Th_) and the second value 20 V above, show *n*-type characteristics including saturation of the monoadduct transistor, whereas no current was detected for both hexa-adduct films.

**Figure 4:**
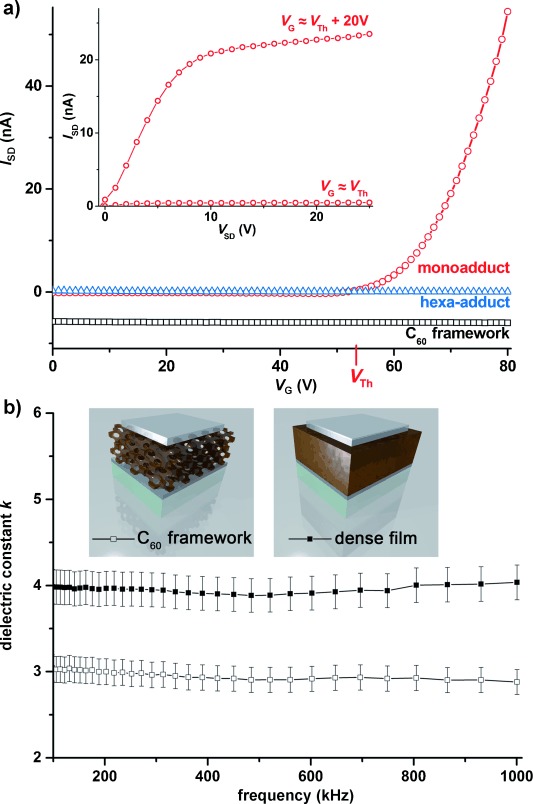
Characterization of the electronic properties by field-effect mobility and impedance measurements. a) Field-effect measurements of three different samples in thin-film transistor geometry. The main graph shows transconductance curves of a monoadduct (○), a hexa-adduct without silane groups (▵), and a C_60_-framework film (□) at *V*_SD_=20 V. The *V*_Th_ value refers to the monoadduct. The data of the C_60_ framework are shifted by −6 nA for clarity. Inset in (a): conductance curves of the monoadduct-based device. b) Dielectric constant values over a broad frequency range obtained by impedance measurements on sandwich-type devices (see inset). By introducing mesoporosity, the dielectric constant for the extracted fullerene-framework film is lowered by 1.0 compared to a dense fullerene film.

In conclusion, the measurements show that the modification of C_60_ with six side chains causes the electron mobility to decrease drastically. Compared to the electron mobility of C_60_ which is of the order of 1–2.5 cm^2^ V^−1^ s^−1^,[17] the value for the monoadduct is already decreased by four orders of magnitude. This observation is consistent with previous studies of mobilities in fullerene derivatives, which show that the mobility decreases with increasing distance between the fullerenes.[18]

The electronic properties of a material are not only defined by its electron mobility, which characterizes the velocity of electrons inside the material in response to an electric field, but also by the dielectric constant *k*, which relates to the polarizability of the material. Impedance measurements made with sandwich-type devices containing dense films of the hydrolyzed precursor give *k* values of around 4 (Figure 4 b). In contrast to this, theoretical studies predict that with a network of fullerene cores connected by various linkers ultra-low *k* values, even below 2, can be achieved.[19] The structure of the linking side chains we used is more complex than the ones used for the calculations, which were shorter and sometimes only alkyl chains. We assume that the higher content of C=O and Si=O bonds present in our precursor yields a higher polarizability and lower porosity and therefore a higher dielectric constant.[20] For example, the structure includes twelve siloxane groups per fullerene core, which is two times more than assumed in the study by Hermann et al.^[19a]^ We observe that the *k* value for the porous compared to the dense fullerene-framework film is reduced to around 3 (Figure 4 b).

We have demonstrated the synthesis of a new hexafunctionalized C_60_ adduct with octahedral symmetry, which was employed as a building block to create the first example of a highly-ordered three-dimensional covalent fullerene framework. Thin films of this material were synthesized by block copolymer template-directed, evaporation-induced self-assembly, resulting in a periodic orthorhombic structure with *Fmmm* symmetry that, after template removal, revealed a pore size of around 7.5 nm. As a result of the functionalization of the fullerene, the resulting material has greatly differing electronic properties compared to pristine C_60_. The dielectric constant of the porous fullerene framework was decreased relative to its dense analogue showing values of around 3 and 4, respectively, over a wide frequency range. It is envisioned that the method of template-directed self-assembly of a surface-functionalized C_60_ to make a highly-ordered 3D covalent fullerene framework could in principle be extended to other organic molecules. For example, the method could be employed to prepare periodic porous conjugated polymer and covalent organic framework[21] analogues with potential applications in areas such as catalysis, gas storage, and drug release.

## Experimental Section

The detailed synthetic procedures for the modified fullerene precursors as well as characterization can be found in the Supporting Information.

Fullerene-framework films were synthesized by adding HCl (6 μL of a 0.2 m solution) to a solution of Pluronic F127 (12 mg, 0.95 μmol) in ethanol (200 μL). This surfactant solution together with the precursor C_60_R_6_ (**3**; 18.9 mg, 5.0 μmol) in ethanol (200 μL) was stirred at room temperature for 3 h. The aged solution was used to spin coat glass and indium tin oxide (ITO) substrates at various speeds from 500 to 1000 rpm. The dried films were heat treated for 18 h at 100 °C. The surfactant template was extracted into ethanol under reflux for 10 h in four cycles with fresh solvent for each cycle.

Dedicated to Dr. Klaus Römer on the occasion of his 75th birthday
