# Supplementary material for: A Highly-Ordered 3D Covalent Fullerene Framework
Source: Angew Chem Int Ed Engl. 2015 May 8;54(26):7577–81. doi: 10.1002/anie.201411344 (PMC4510781; doi:10.1002/anie.201411344)
Supplement: Supplementary file 1 [file anie0054-7577-sd1.pdf]

Angewandte Chemie
Eine Zeitschrift der Gesellschaft Deutscher Chemiker

Supporting Information

A Highly-Ordered 3D Covalent Fullerene Framework**

*Norma K. Minar, Kun Hou, Christian Westermeier, Markus Döblinger, Jörg Schuster, Fabian C. Hanusch, Bert Nickel, Geoffrey A. Ozin, and Thomas Bein**

anie_201411344_sm_miscellaneous_information.pdf

Table of contents:

1 General Experimental Information **S1**

2 Precursor Synthesis and Characterization **S2**

3 C_{60}-framework Synthesis **S4**

4 Solid-state NMR of the C60-framework **S5**

5 Structure Determination **S6**

6 Thermal Stability **S7**

7 Determination of Optical and Electronic Properties **S7**

1 General Experimental Information

Chemicals: Fullerene (C_{60}) was purchased from BuckyUSA (99.5 % grade). 9,10-Dimethylanthracene (DMA, >98 %) was obtained from Tokyo Chemical Industry. Platinum-divinyltetramethyldisiloxane complex (Karstedt catalyst, 3–3.5 % Pt in vinyl terminated polydimethylsiloxane) was acquired from abcr GmbH&Co.KG. All other chemicals and solvents were purchased from Aldrich and used without further purification.

Characterization: Small angle X-ray diffraction measurements were performed on a Bruker D8 Discover with Ni-filtered CuK_α radiation (0.154 nm) and a position-sensitive detector (LynxEye). TEM images were acquired with a FEI Titan 80–300 microscope equipped with a field emission gun operated at 300 kV. The fullerene framework material was removed from the glass substrate and deposited on a carbon-coated copper grid for imaging. The SEM images were recorded with a Jeol 6500F field emission scanning electron microscope at 3 to 5 kV. Nitrogen sorption isotherms were measured with a Quantachrome Nova 4000e instrument at −196 °C. Prior to the measurements, all samples were degassed at 120 °C for 5 h. Brunauer Emmett Teller (BET) surface areas were calculated from the linear section of the BET plot (p/p_0 = 0.05–0.2). The pore-size distribution and pore volume were determined using a quenched solid state density functional theory (QSDFT) method (DFT kernel used: N_2 at −196 °C on carbon, cylindrical and spherical pores for the adsorption branch). For liquid NMR characterization of the observed signal multiplicities the following abbreviations were used: s (singlet), d (doublet), t (triplet) and m (multiplet). ^{29}Si magic-angle spinning-(MAS) and ^{13}C cross polarization(CP)-MAS-NMR spectra were recorded on a Bruker Avance III 500 spectrometer (11.7 T, at 99.4 MHz for ^{29}Si and 125.8 MHz for ^{13}C), using a sample spinning frequency of 10 kHz. The UV-Vis absorption spectrum of the precursor solution and the fullerene framework film coated on a quartz slide was recorded with a Hitachi U-3501 spectrometer in transmission. The infrared measurements were carried out in transmission with a Bruker-Equinox 55 spectrometer, with the precursor or removed film material pressed into a pellet with potassium bromide. Thermogravimetric (TG) measurements were

performed in a stream of synthetic air or nitrogen (25 mL min^{-1}) on a Netzsch STA 440 C TG/DSC. The measurements were carried out with a heating rate of 10 K min^{-1} and a temperature range from 30 °C to 900 °C. The capacitance was measured with a N4L PSM 1700 PsimetriQ frequency response analyser with an LCR active head. For frequencies above 100 kHz a shunt resistor of 100 Ω was chosen. The data were evaluated with the PSMcomm software, version 1.3.1.211.

2 Precursor Synthesis and Characterization

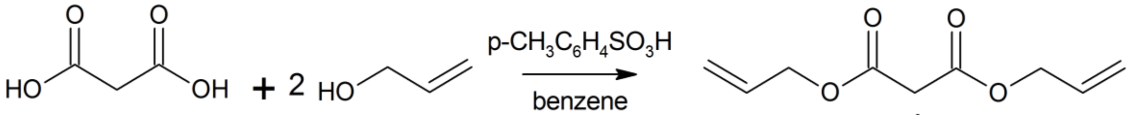

Scheme S1. Reaction scheme for the synthesis of diallyl malonate 1.

Diallyl Malonate 1[1]: Malonic acid (1.0 g, 10 mmol), allyl alcohol (3.0 mL, 44 mmol) and *p*-toluenesulfonic acid (0.11 g, 0.64 mmol) were dissolved in 50 mL benzene. The water resulting from the esterification reaction was removed azeotropically using a Dean-Stark apparatus. After 6 h the apparatus was cooled down to ambient temperature. 50 mL diethyl ether was added to the reaction solution and the organic phase was washed with aqueous saturated $NaHCO_3$ (2x15 mL), brine (1x15 mL) and dried over $NaSO_4$. After removing the solvent under reduced pressure a yellow oil remained. This crude product was purified by flash chromatography on a silica column (60A, 0.035–0.070 mm) with ethyl acetate/ hexane (1:10), to obtain the pure product as light yellow oil (1.7 g, 92 % yield). The NMR data correspond very well with the literature.[1]

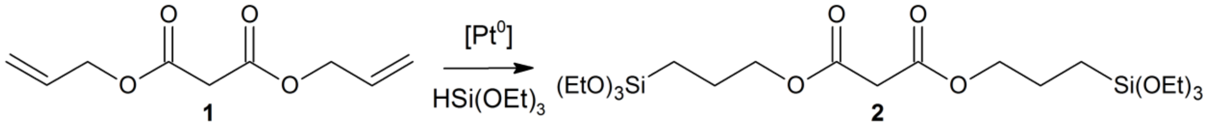

Scheme S2. Reaction scheme for the synthesis of diallyl malonate siloxane 2.

Diallyl Malonate Siloxane 2[2]: 0.78 g of **1** (4.2 mmol) was dissolved in THF (5 mL) in nitrogen atmosphere. To this solution triethoxysilane (2.3 mL, 12 mmol) and Karstedt catalyst (20 µL, 0.15–0.18 M) were added. The mixture was stirred for 12 h at room temperature and in nitrogen atmosphere. Subsequently the solvent and the excess triethoxysilane were evaporated under vacuum to give compound **2**.

^{1}H NMR (270 MHz, $CDCl_3$, TMS): δ = 4.08 (t, J = 8 Hz, 4H), 3.79 (t, J = 8 Hz, 12H), 3.32 (s, 2H), 1.72 (m, J = 8 Hz, 4H), 1.18 (t, J = 5.4 Hz, 18H), 0.59 (t, J = 8 Hz, 4H) ppm.

^{13}C NMR (270 MHz, $CDCl_3$, TMS): δ = 166.67(C=O), 67.53, 58.46, 41.60, 22.13, 18.30, 7.13 ppm.

MS (EI, positive ion, m/z) $[M-OC_2H_5]^+$ calculated for $C_{19}H_{39}O_9Si_2$, 467.21 , found 467.3.

Hexakis-adduct $C_{66}(COOC_3H_6SiO_3Et_3)_{12}$ 3 ($C_{60}R_6$)[3]: Fullerene C_{60} (0.20 g, 0.28 mmol) and 9,10- dimethylanthracene (DMA, 0.572 g, 2.8 mmol) were dissolved in 120 mL toluene and stirred at room temperature under nitrogen and exclusion of light for 3 h. After the addition of tetrabromomethane (9.2 g, 28 mmol) and compound **2** (1.4 g, 2.8 mmol) the mixture was stirred for another 1 h and then 1,8-diazabicycloundec-7-ene (DBU, 0.83 mL, 5.5 mmol) was added. The reaction solution was stirred at room temperature and under nitrogen for 4 days. The precipitate of the reaction was separated by centrifugation. The purification of **3** was achieved by flash chromatography on a silica column (60A, 0.035–0.070 mm) using first anhydrous dichloromethane and then dichloromethane/ ethanol (20:1) followed by dichloromethane/ ethanol (10:1) as the eluent solutions. The C_{60} silane precursor **3** was obtained as a red solid at a yield of 40 % (80 mg).

Compound **3** was stored in dry dichloromethane solution under argon for further use. The molecular structure of **3** was determined by combining ^{13}C, ^{29}Si NMR and IR data. Unambiguous evidence for the formation of the precursor compound **3** was provided by ^{13}C NMR spectroscopy (Figure S1). The two resonances observed for the sp^2 hybridized fullerene core located at 145.84 and 141.12 ppm prove a T_h-symmetric addition pattern surrounding the fullerene core.[4] A signal for the bridgehead C atom (δ = 45.3 ppm), a signal for the carbonyl group (δ = 163.7 ppm), and three signals for the $(CH_2)_3$ linker (δ = 6.38, 22.06, 68.89 ppm) were also observed (Figure S1).

^1H NMR (270 MHz, $CDCl_3$, TMS): δ = 4.19 (t, $COOC\mathbf{H}_2CH_2CH_2$, 24H), 3.77 (m, $OC\mathbf{H}_2CH_3$, 72H), 1.79 (m, $CH_2C\mathbf{H}_2CH_2$, 24H), 1.18 (t, $OCH_2C\mathbf{H}_3$, 108H), 0.61(m, $CH_2CH_2C\mathbf{H}_2Si$, 24H) ppm.

^{13}C NMR (270 MHz, $CDCl_3$, TMS): δ = 163.71 (C=O), 145.84, 141.12 (C_{60}, sp^2), 68.89 (C_{60}, sp^3), 68.78 ($COO\mathbf{C}H_2CH_2CH_2$,) 58.80 ($O\mathbf{C}H_2CH_3$), 45.27 (O=C**C**C=O), 22.06 ($CH_2\mathbf{C}H_2CH_2Si$), 18.43 ($OCH_2\mathbf{C}H_3$), 6.38 ($CH_2CH_2\mathbf{C}H_2Si$) ppm.

^{29}Si NMR (270 MHz, $CDCl_3$, TMS): δ = −45.77 ppm.

IR (KBr): v (in cm^{-1}) = 2975, 2926, 2890 ($v_{C\text{-}H}$), 1748 ($v_{C=O}$), 1444, 1391, 1230 ($v_{C\text{-}O\text{-}C}$), 1103, 1078 ($v_{Si\text{-}O\text{-}C}$), 954 ($v_{asym\ Si\text{-}O\text{-}C}$), 784.

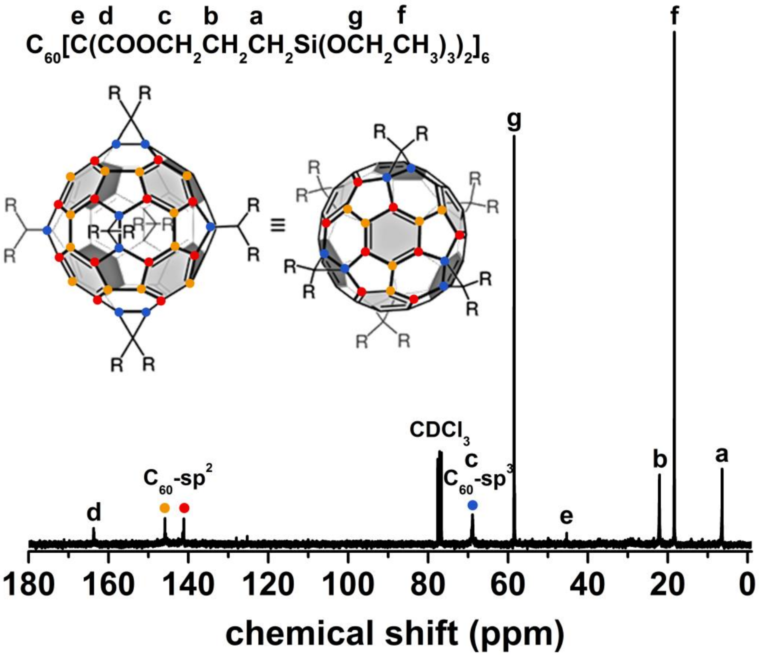

Figure S1. ^{13}**C-NMR of the isolated hexakis-adduct 3.** All signals can be assigned to the carbon atoms of **3**. The 3 different chemical environments resulting from a symmetrical octahedral addition pattern at the C_{60} core are marked with a color code in the spectra and on the molecule scheme. R = $COO(C_3H_6)Si(OEt)_3$.

3 C_{60}-framework Synthesis

In a typical synthesis, 12 mg of Pluronic® F127 (0.95 µmol) was dissolved in 200 µl of ethanol followed by addition of 6 µL of 0.2 M HCl to form a surfactant solution. To this solution, 18.9 mg of precursor **3** $C_{60}R_6$ (5 µmol) in 200 µl ethanol solution was added and the mixture was stirred at room temperature for 3 h. The aged solution was used to spin-coat glass and ITO substrates at various speeds from 500 to 1000 rpm. This way, brown, homogeneous and smooth films were obtained (Figure S2). Bulk thick films were obtained by casting the coating solution into a petri dish and aging in ambient air for 5 days. To remove the surfactant, the cast films were solvent extracted four times with ethanol at 70 °C. For each cycle of extraction, the material was left in EtOH for 10 h. Before solvent extraction, the material was thermally treated at 100 °C in nitrogen for 18 h to stabilize the framework before template removal. Bulk material was extracted in the same way, but the material was removed from the glass substrates beforehand.

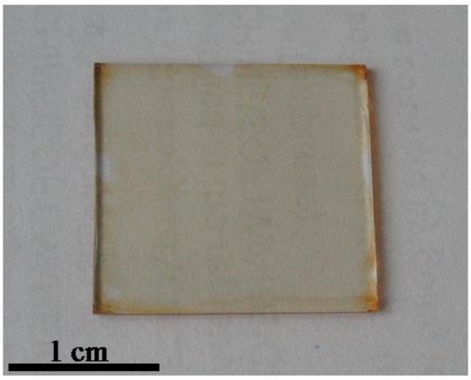

Figure S2. Photograph of the spin-coated porous C_{60}-framework film.

4 Solid-state NMR of the C60-framework

The local chemical structure of the fullerene framework was examined by 13C and 29Si solid-state NMR spectroscopy (see Figure S3). The 13C cross polarization (CP) - magic-angle spinning (MAS) - NMR of the film removed from the substrate corresponds very well with that of the fullerene precursor, which confirms the integrity of the molecular structure of the precursor in the fullerene framework (compare Figures S1 and S3 A). The signals at δ = 8.7 and 22.0 ppm can be assigned to the CH_2 groups connected directly to and next to the silicon atom (Figure S3 A, label a, b). These signals correspond to the ^{13}C signals at δ = 6.38 and 22.1 ppm of the fullerene precursor molecule (see Figure S1). The slight shift of the signal of the carbon atoms adjacent to the Si atoms is due to the hydrolysis of the ethoxy groups (followed by condensation), which changes the chemical environment of the silicon atom. The signal at 46.1 ppm is assigned to the malonate carbon atoms anchored on the fullerene core (Figure S3 A, label e), which is in good agreement with the corresponding signal in the precursor. The signal at 69.6 ppm is a result of the overlapping signals of the sp^3 hybridized carbon of the C_{60} and the carbon atoms of the propyl chain connected to the carbonyl via oxygen, and matches also with the corresponding signal in the precursor (Figure S1, label c, δ = 68.9 ppm). The broad signal centered at around 143 ppm can be unambiguously assigned to the sp^2 fullerene core, while the signal at 162.8 ppm is assigned to the carbonyl (C=O) group of the ester (Figure S3 A, label d). The ^{13}C-CP-MAS-NMR also proves that the ester group in the precursor is robust enough to survive the synthesis conditions. In the solid state ^{29}Si MAS-NMR spectrum (Figure S3 B), resonances at −49.3, −58.2 and −67.7 ppm are representative of T-type organosilica species [Tn=RSi$(OSi)_n(OH)_{3-n}$], which can be assigned to T1, T2, T3 units of the condensed silsesquioxane moieties, respectively. The clear absence of Q units [Qn=Si$(OSi)_n(OH)_{4-n}$] at around −100 ppm shows that there is negligible hydrolytic Si-C bond cleavage and that the siloxane-bridged organic linkers are maintained intact in the fullerene framework under the synthetic conditions. The ^{29}Si MAS-NMR spectrum in Figure S3 C proves that the Si-C bond cleavage is negligible

after the 300 °C thermal treatment because very little new intensity is detected in the chemical shift range around −100 ppm for Si-Q-species.

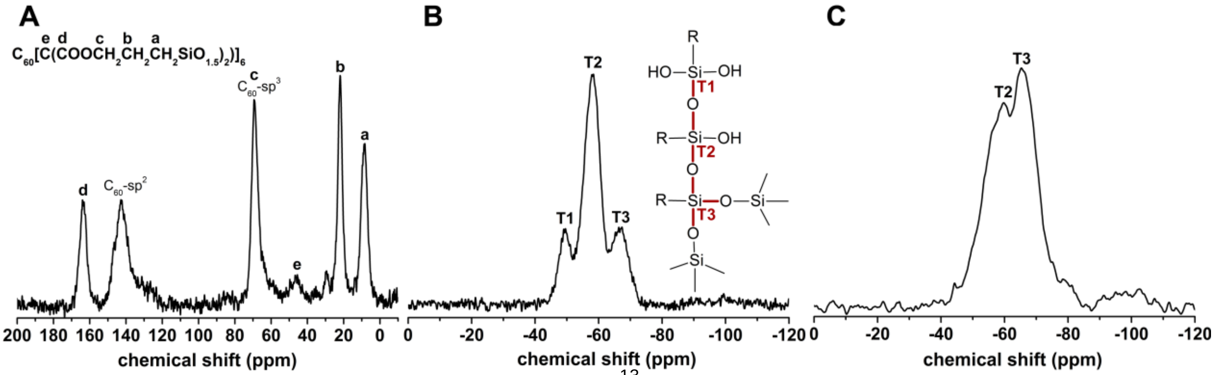

Figure S3. ssNMR of the fullerene framework. A: ^{13}C CP-MAS-ssNMR of solvent extracted fullerene framework. B: ^{29}Si MAS-ssNMR of solvent extracted fullerene framework. C: ^{29}Si MAS-ssNMR of solvent extracted fullerene framework heated at 300 °C in N_2.

5 Structure Determination

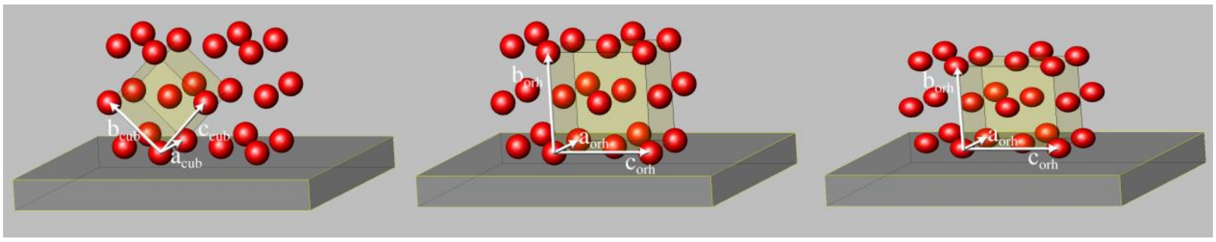

Figure S4. Schematic picture of the relationship of the mesoporous structure in the cubic and the orthorhombic setting with respect to the substrate surface. On the left side, the initial cubic *Im-3m* structure is depicted, with $[011]_{cub}$ along the substrate normal. The image in the middle shows the initial structure described in an orthorhombic setting with *Fmmm* symmetry and $[010]_{orh}$ along the substrate normal. The lattice basis vectors change as follows: $a_{orh} = a_{cub}$, $b_{orh} = (b_{cub}+c_{cub})$, and $c_{orh} = c_{cub}-b_{cub}$. On the right side the structure is shown after shrinkage: a_{orh} and c_{orh} remain constant, while b_{orh} is decreased.

6 Thermal Stability

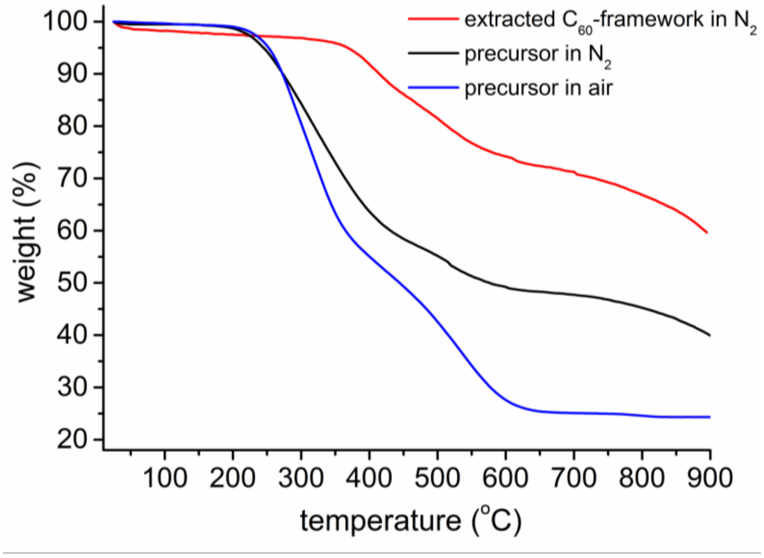

Figure S5. Thermogravimetric curves of precursor and solvent extracted fullerene framework under nitrogen or air.

7 Determination of Optical and Electronic Properties

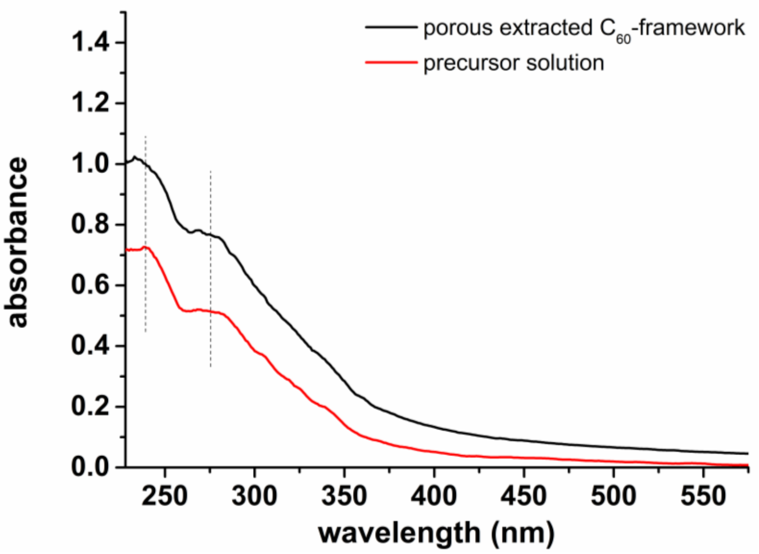

Figure S6. UV-Vis spectra of a fullerene framework film on fused silica substrate and the corresponding precursor 3 in acetonitrile solution (10^{-4} M). The absorption maxima of the C_{60}-framework show no bathochromic shift compared to the molecule in solution. Therefore no electronic coupling or aggregation occurs in the framework.

TFT and Sandwich Type Devices

For thin-film transistor measurements a highly p-doped silicon substrate (Silicon Materials, 1–30 Ohm cm) was used as the gate contact and the dielectric was a SiO_2/Si_3N_4 bilayer with a thickness of 100 nm each. 50 nm thick aluminium contacts were thermally evaporated through a shadow mask with a channel width of 2 mm and a channel length of 50 µm on top of the respective active material, which was spin-coated from solution before. The fullerene framework was heat treated and solvent extracted before evaporation of the top contacts, as described earlier. The transistor devices were electrically contacted with a point probe station and device characteristics were measured under ambient conditions with a Keithley Instruments Source Meter 2612.

Devices for capacitance measurements were built with a sandwich-type layout. Dense films of the hydrolyzed precursor and films of the porous fullerene framework were spin-coated onto a tin-doped indium oxide (ITO) coated glass (VisionTek, 150 nm ITO, 12–15 Ω/□). The films were heat treated for 18 h at 100 °C. The porous fullerene framework films were solvent extracted as mentioned above. 80 nm thick aluminium contacts were evaporated on top of the films with a shadow mask to give a final device area of 0.09 mm².

The film thickness was determined from SEM cross section images (see Figure S7).

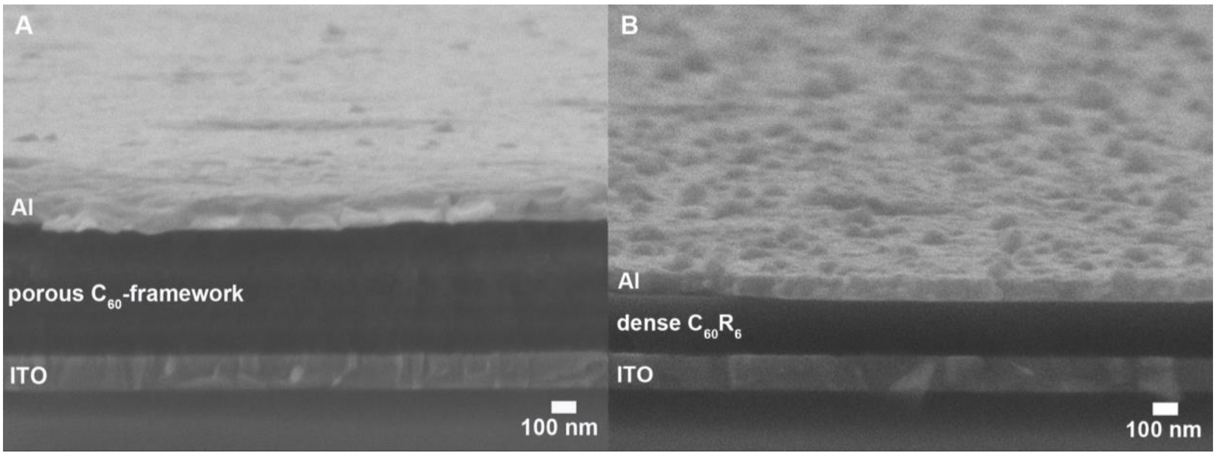

Figure S7. SEM cross section images of sandwich-type devices for capacitance measurements. A: Device with porous C_{60}-framework film. **B:** Device with a dense film of hydrolyzed C_{60}-hexakis adduct. All layers are labeled within the images.

References

[1] R. Jana, R. Trivedi, J. A. Tunge, *Organic Letters* **2009**, *11*, 3434-3436.

[2] D. N. Willing, Vol. US3419593, US, **1968**, p. 14 pp.

[3] X. Camps, A. Hirsch, *J. Chem. Soc., Perkin Trans. 1* **1997**, 1595-1596.

[4] a) P. Witte, F. Hoermann, A. Hirsch, *Chem.--Eur. J.* **2009**, *15*, 7423-7433; b) A. Hirsch, I. Lamparth, T. Groesser, H. R. Karfunkel, *J. Am. Chem. Soc.* **1994**, *116*, 9385-9386.
